# The correlates of hyperechogenicity of the substantia nigra in subjects at risk of dementia with Lewy bodies

**DOI:** 10.1007/s00702-026-03171-7

**Published:** 2026-05-14

**Authors:** Kateřina Zelníčková, David Ondráček, Marek Mikulec, Jiří Mekyska, Jan Farkaš, Martin Lamoš, Irena Rektorová

**Affiliations:** 1 Applied Neuroscience Research Group, Brain and Mind Research Program, Central European Institute of Technology (CEITEC), Masaryk University, Kamenice 753/5, 625 00 Brno, Czech Republic; 2 Faculty of Medicine, Masaryk University, Kamenice 753/5, 625 00 Brno, Czech Republic; 3 First Department of Neurology, Faculty of Medicine, St. Anne’s University Hospital, Masaryk University, Pekařská 664/53, 656 91 Brno, Czech Republic; 4 Department of Telecommunications, Brno University of Technology, Technická 12, 616 00 Brno, Czech Republic

**Keywords:** transcranial sonography, Substantia nigra, Prodromal dementia with Lewy bodies, EEG, Cognitive, Sex differences

## Abstract

Substantia nigra hyperechogenicity (SN+) detected by transcranial sonography (TCS) has been proposed as a risk marker for synucleinopathies, particularly Parkinson’s disease. Its role in prodromal dementia with Lewy bodies (DLB) remains insufficiently explored. To examine clinical, cognitive, genetic, EEG, and plasma biomarker differences between SN+ and normal echogenicity (SN−) individuals in a well-characterized at-risk-of-DLB cohort, with additional sex-stratified analyses. A total of 121 participants aged 55–70 years from an at-risk-of-DLB cohort underwent TCS, neurological and neuropsychological assessment, nighttime wrist actigraphy, high-density EEG, genetic testing for APOE allelic variants, and plasma biomarker analysis (NfL, GFAP, pTau181). SN status was defined using laboratory-specific cut-offs. Group differences were assessed using non-parametric statistics with relevant corrections for multiple comparisons and adjustment for age. SN+ status as compared to SN− status was associated with poorer verbal memory performance and altered visuo-constructive performance (driven mostly by males), and significant differences in distinct actigraphy-derived sleep parameters. SN+ participants showed higher plasma NfL levels (particularly in males) and a higher prevalence of DLB-specific EEG patterns (driven by females). No significant associations were observed for global cognition, motor parkinsonism, cognitive fluctuations, hallucinations, REM sleep behavior disorder, depressive symptoms, olfactory function, plasma biomarkers of degeneration, neuroinflammation, β-amyloid deposition, or APOE status. In individuals at risk of DLB, SN hyperechogenicity is linked to early markers of neurodegeneration and cholinergic dysfunction, including elevated NfL and DLB-specific EEG abnormalities, as well as selective posterior cortical cognitive impairment and sleep disturbances. The observed sex-specific effects require further investigation.

## Introduction

Transcranial sonography (TCS) is an inexpensive, widely available, and non-invasive screening method for Parkinson’s disease (PD) (Berg 2011; Mijajlovic et al. 2014); hyperechogenicity of the substantia nigra (SN) has been suggested as a risk marker for prodromal PD (Heinzel et al. 2019). Regarding dementia with Lewy bodies (DLB), TCS showed a sensitivity of 88.9% and specificity of 81.2% for distinguishing DLB from Alzheimer’s disease (AD) (Favaretto et al. 2016). Combining the echogenicity of the SN and widening of the frontal horns of the lateral ventricles could predict a diagnosis of early DLB (Planas-Ballvé et al. 2024). SN hyperechogenicity (SN+) may be related to age and gender, with a more positive correlation between greater SN echogenicity and PD duration in men than in women (Zhou et al. 2018), although these results were not supported by other studies (Pedroso et al. 2013; Wei et al. 2023). While most works correlate SN echogenicity with motor and nonmotor symptoms in fully developed PD, few studies have explored echogenicity in healthy elderly subjects or those at risk of synucleinopathies. For example, more subtle motor, olfactory, and cognitive deficits were found in SN+ than in SN− healthy individuals (Liepelt et al. 2008; Hoeppner et al. 2009; Behnke et al. 2010; Berg et al. 2010; Yilmaz et al. 2016a, b). Premotor SN+ subjects were more frequently associated with hyposmia, slight motor parkinsonian signs, male gender, and positive family history of PD (Liepelt et al. 2011).

We examined 139 subjects at risk of DLB from a prodromal DLB cohort described in our previous works (Železníková et al. 2025; Mitterová et al. 2025; Onofrj et al. 2026) who underwent TCS examination. The subjects were recruited via a questionnaire and assessed using neurological examination, validated clinical scales and questionnaires, cognitive testing using a detailed cognitive battery, high-density (HD) EEG, and digital markers examining cognitive symptoms and sleep abnormalities. Plasma biomarkers of neurodegeneration, neuroinflammation, and β-amyloid pathology as well as APOE allelic variants, were also examined (see the Methods section for details).

Our aim was to evaluate differences in SN+ and SN − in this well-described at-risk-of-DLB cohort while controlling for age. Because of the described sex-related differences in TCS, we additionally explored the differences separately in males and females. We specifically focused on core clinical features of DLB including parkinsonism, cognitive fluctuations, hallucinations, and REM sleep behavior disorder (RBD). Based on previous research, visuo-constructive tests and verbal fluency are particularly relevant for differentiating DLB from AD. A similar finding is evident when comparing prodromal DLB and PD. Overall, attentional, executive, and visuo-constructive tests appear to be the best predictors of DLB even in prodromal phases (Kemp et al. 2017; Wyman-Chick et al. 2025a). Notably, memory impairment was also common in prodromal DLB (> 70%), although it was associated with a higher risk for AD diagnosis (Ting et al. 2023).

We therefore further focused on cognitive deficits in attention/executive functions, verbal fluency, visuo-constructive functions, and both visual and verbal memory. Depressive symptoms, other sleep-related digital measures, and olfactory deficits were also assessed. As a novelty, we evaluated EEG compressed spectral array (CSA) patterns based on the works of Bonanni et al. (2008, 2015, 2016), Mahajan et al. (2025) and examined whether DLB-specific EEG patterns were related to SN echogenicity. The CSA is a depiction of frequency and power change over time. It represents the epoch-to-epoch analysis of EEG power density spectra by fast Fourier transform for each electrode, which indicates peaks of resting-state EEG (rsEEG) power density values (Mahajan et al. 2025). The utility of the characterization of EEG patterns stems from the fact that the spatial and frequency content of the rsEEG power density spectra changes with time in relation to the changes in the participant’s vigilance level and correlates with cognitive fluctuations in DLB (Bonanni et al. 2008). Finally, we also assessed how plasma biomarkers of axonal loss, neuroinflammation, and β-amyloid deposition as well as APOE status relate to TCS results.

We hypothesized that the SN+ group is more associated than the SN− group with pronounced core clinical features of DLB, olfactory loss, and lower cognitive performance in tests evaluating attention/ executive functions including verbal fluency, visuo-constructive functions, and memory, and with clinical differences more pronounced in males than in females. We also hypothesized that DLB-related EEG patterns are more prevalent in the SN+ group than in the SN− group. Finally, we hypothesized that SN+ is related to both plasma markers of neurodegeneration (NfL) and neuroinflammation (GFAP), and that it is not related to the marker of brain β-amyloid deposition (pTau181).

## Methods

### At-risk-of-DLB cohort

Volunteers aged 55–70 were recruited based on a screening questionnaire asking about the presence of RBD, reduced arm swing, bradykinesia or rigidity, hypokinetic dysarthria, cognitive deficits and cognitive fluctuation, chronic constipation, hyposmia, excessive daytime sleepiness, mood disorders, and visual hallucinations/ illusions. An on-site visit of the pre-screened individuals included an objective neurological examination and medical history, olfactory examination (University of Pennsylvania Smell Identification Test; UPSIT) (Doty et al. 1984), examination for motor symptoms of parkinsonism (Unified Parkinson’s Disease Rating Scale; UPDRS III) (Movement Disorder Society Task Force on Rating Scales for Parkinson’s Disease 2003), examination for symptoms of depression (Geriatric Depression Scale; GDS) (Greenberg 2007), global cognitive examination (Montreal Cognitive Assessment; MoCA) (Nasreddine et al. 2005), detailed cognitive testing using a comprehensive battery of tests examining all cognitive domains, assessment of RBD using a screening questionnaire (RBDSQ) (Stiasny-Kolster et al. 2007), assessment of sleep disturbances by digital markers using nighttime wrist actigraphy, examination for fluctuations in attention (Mayo Fluctuations Scale; MFS) (Ferman et al. 2004), and assessment of hallucinations using the Neuropsychiatric Inventory (NPI—hallucinations subscore) questionnaire (Cummings et al. 1994). In addition, all participants underwent a five-minute resting-state HD EEG recording (256-channels) with closed eyes, and blood sampling for plasma biomarkers of neurodegeneration and APOE testing.

Subjects were classified as having MCI if they had subjective cognitive complaints and objective cognitive impairment on neuropsychological testing, defined as performance at least 1 SD below normative values in two or more tests, or at least 2 SD below normative values in one test from the neuropsychological battery (Aarsland et al. 2010). Based on the clinical and cognitive examinations, subjects were classified into four groups: (1) Subjective complaints but healthy on objective examination, further referred to as healthy controls (HC), with no core clinical features (CCF) of DLB (McKeith et al. 2017) and without mild cognitive impairment (MCI); (2) presence of 1–2 CCF but no MCI (CCF+, MCI−); (3) CCF + and MCI+, further referred to as MCI with Lewy bodies (MCI-LB) (McKeith et al. 2020); and (4) a small subset of participants met the criteria for mild cognitive impairment (MCI) without core clinical features of DLB (CCF−, MCI+).

Of 139 at-risk-of-DLB subjects who also underwent TCS, 121 participants had sufficient temporal bone window quality. Altogether 25 subjects were tested as HC (CCF−, MCI−), 46 individuals were tested as CCF+, MCI−, 37 subjects were diagnosed as MCI-LB (CCF+, MCI+), and 13 subjects as MCI without CCF (CCF−, MCI+). All subjects were dopaminergic and cholinergic medication naive, and they each signed an informed consent form. The study was approved by the local ethics committee.

### Investigation by the method of transcranial sonography

The patients were examined in a supine position. A temporal window was used; the transducer was placed on the temple parallel to the orbitomeatal line. Based on recommendations published elsewhere (Walter and Školoudík 2014) we investigated with a 14–16 cm image depth, a dynamic range of 45–55 dB, and a center frequency of insonation from 2.0 to 3.5 MHz. The examination of SN was performed from both sides; echogenicity was measured only in the ipsilateral SN with respect to the insonated side (Walter and Školoudík 2014). A butterfly-shaped hypoechoic structure of mesencephalon is visible in the plane of the midbrain, bordered by hyperechogenic basal cisterns (Mijajlovic et al. 2014). In accordance with the recommendations, a planimetric measurement was used; the echogenic signal of the SN was manually encircled and then the echogenic area was automatically calculated by an ultrasound device (Walter and Školoudík 2014). For statistical analyses, when bilateral measurements were available the higher value of SN echogenicity was used. Our laboratory-established cut-off values for the TCS of SN were determined using the 90th and 75th percentiles. For the purposes of this study, we define an echogenic area of ≥ 0.2 cm^2^ as clearly hyperechogenic, an echogenic area of 0.16–0.19 cm^2^ as a mildly hyperechogenic, and an area of ≤ 0.15 cm^2^ as clearly negative. These values are in accordance with the literature (Walter and Školoudík 2014).

### Neuropsychological battery

The neuropsychological battery used to identify MCI subjects included an evaluation of visuospatial memory using the Brief Visuospatial Memory Test (BVMT) (Benedict 1997). Verbal memory was evaluated with the Philadelphia Verbal Learning Test (PVLT), including immediate recall, short-delay recall, and long-delay recall (Bezdicek et al. 2014). Visuospatial abilities were examined using the Judgment of Line Orientation (JLO) test (Nikolai et al. 2015). Semantic and lexical verbal fluency tasks were administered to assess language function (Nikolai et al. 2015); attention and executive functioning were evaluated using the Digit Symbol Substitution Test (DSST), Picture Arrangement and Letter–Number Sequencing tasks (Taub et al. 2004). For the purpose of the current study and based on previous literature (Kemp et al. 2017; Ting et al. 2023; Wyman-Chick et al. 2025a), we focused on attention/ executive function (DSST, Letter–Number Sequencing and Picture Arrangement); visuo-constructive functions were assessed by the digitized pentagon copying test (PCT); semantic and lexical verbal fluency (language function), and verbal (PVLT) and visual (BVMT) memory.

### Digitized pentagon copying test (PCT)

Participants performed the PCT on an A4 sheet of paper placed on and fixed to a Wacom Intuos 4 M digitizing tablet (Wacom Co., Ltd., Japan). The drawing process was recorded at a sampling frequency of 133 Hz. The acquired data consisted of a time series of horizontal and vertical pen coordinates, corresponding timestamps, and a binary pen-state variable indicating in-air movement, coded as 0 and recorded up to 1.5 cm above the tablet surface, and on-surface movement, coded as 1. Based on the segmented on-surface trajectories, Shannon entropy was computed to quantify movement irregularity. This entropy-based feature quantified the smoothness and stability of line execution during drawing (Brabenec et al. 2022).

## Sleep assessment

Sleep was objectively assessed using wrist actigraphy. All participants underwent continuous 7-day monitoring with the GENEActiv Original device (ActivInsights Ltd., UK), worn on the non-dominant wrist. The device recorded tri-axial acceleration signals throughout the monitoring period. Raw actigraphy data were preprocessed to identify sleep and wake intervals using the algorithm described by Mikulec et al. (2022). Based on the resulting sleep–wake classification, the following nocturnal sleep parameters were extracted for each night: total sleep time, defined as the total duration of sleep during the primary nocturnal sleep episode; number of awakenings longer than 5 min; sleep efficiency, calculated as the ratio of total sleep time to time in bed; and sleep fragmentation index, defined as the number of awakenings relative to total sleep time. For subsequent statistical analyses, nightly values of each parameter were averaged across the 7-day monitoring period to obtain a single representative value per participant.

### EEG patterns

All subjects underwent a scalp HD-EEG recording using the 256-channel EEG system EGI GES 400 MR (Electrical Geodesics, Inc.) with sampling frequency of 1 kHz. Participants were asked to stay still and sit with eyes closed in the monitoring seat in a Faraday-shielded room and five minutes of resting-state data were recorded. HydroCel GSN 220MR cap with Cz reference electrode was used. Impedances of all channels were held below 50 kΩ based on EEG manufacturer’s recommendations.

EEGLAB and MATLAB R2023a (The MathWorks, Inc, Natick, USA) were used for processing the EEG data. The number of channels was reduced to 204, discarding neck line and face electrodes. The data were filtered to 1–40 Hz by 2nd order Butterworth filter. The filtration was performed in forward and reverse directions for zero phase distortion. Bad channels were detected (when they were greater than three standard deviations of the mean of all channels) and interpolated by spheric spline (less than 10 channels). Data segments with Global Field Power (GFP) greater than ten standard deviations of the mean GFP were marked as artifacts. Independent component analysis (ICA) was used to suppress signals related to eye movements and electrocardiogram (ECG). After ICA decomposition, artifact-related components were identified manually by visual inspection. Then, EEG data were back-reconstructed without these components. No more than 10 components were discarded.

EEG patterns are calculated from EEG recordings in posterior scalp electrodes (PO7, PO3, O1, POz, Oz, PO4, O2, PO8). The whole recording is divided into epochs of the same length. The fast Fourier transform (FFT) is used to transform a power-time to a power-frequency spectrum; the dominant frequency, its variability and other variables are then calculated. Altogether six EEG patterns were described (Bonanni et al. 2015). Pattern 1 is observed in healthy people and in people with Alzheimer’s disease (AD), the dominant alpha frequency is above 8 Hz, and the dominant alpha variability is below 0.6 Hz. Patterns 2, 3, 4, and 5 are seen in DLB/ PD-dementia; in pattern 2, the dominant alpha frequency is below 8 Hz and the variability is above 2 Hz; in pattern 3, the dominant frequency is between 5.6 and 7.9 Hz, the variability is below 1 Hz. In pattern 5, the alpha is missing; the pre-alpha frequency is missing in more than two subsequent epochs and the variability is above 4 Hz. Pattern 4 is present in people with Parkinson’s disease dementia (PDD); the dominant alpha frequency is absent, and the dominant frequency is in a spectrum of pre-alpha or theta/delta frequencies, the dominant frequency variability is above 2 Hz.

### Plasma biomarkers

Fasting venous blood samples were collected and plasma biomarkers were analyzed using the Simoa Neuro-2-Plex B toolkit (Quanterix). For the purposes of this study, we included three plasma biomarkers which have been widely studied in prodromal neurodegenerative cohorts, specifically: neurofilament light chain (NfL) reflecting neuroaxonal damage; glial fibrillary acidic protein (GFAP), a marker of astroglial activation; and phosphorylated tau at threonine 181 (pTau181), reflecting tau pathology as well as reportedly showing potential for detecting β-amyloid accumulation (De Meyer et al. 2024; Bolsewig et al. 2024). Plasma concentrations were quantified in picograms per milliliter (pg/mL) by the Clinical Neurochemistry Laboratory, University of Gothenburg (Sweden) in a blinded manner. For all assays, calibrators were run in duplicates and obvious outlier calibrator replicates were masked before curve fitting. Samples were run in single measurements after dilution (4-fold for NfL and GFAP; 2-fold for pTau181), and results were corrected for dilution. Two quality-control (QC) levels were run in duplicates at the beginning and end of each run. We also evaluated APOE4 status. This allele represents a strong, dose-dependent genetic risk factor for Alzheimer’s disease and has been linked also to Lewy body pathology even beyond concomitant AD changes, its pathogenic effect is thought to reflect both a gain of toxic functions and a loss of protective functions (Raulin et al. 2022).

#### Statistical analysis

The normality of all variables was assessed using the Shapiro–Wilk test. As several variables did not meet the assumption of normal distribution, non-parametric statistical methods were applied consistently throughout the analysis.

Although the SN+ and SN− groups did not differ significantly in age according to a two-sample t-test (*p* = 0.1155), age was treated as a potential confounding factor. Therefore, prior to further analyses, the effect of age was regressed out from each variable using linear regression, and all subsequent statistical tests were performed on the resulting residuals. In contrast, gender distribution differed significantly between groups, as indicated by the chi-square test of independence (*p* = 0.0250). Given this imbalance, all primary analyses were performed separately for males and females in addition to the overall cohort analysis.

For each variable, participants were stratified into SN+ and SN− groups, and between-group differences were assessed using the Mann–Whitney U test. These comparisons were conducted for the entire cohort and separately for males and females to account for the significant gender imbalance. To account for multiple comparisons, p-values obtained from actigraphy-derived sleep parameters (total sleep time, awakenings > 5 min, sleep efficiency, sleep fragmentation), BVMT tests, PVLT tests, language measures (lexical and semantic verbal fluency), attention/ executive function tests (DSST, Picture Arrangement and Letter–Number Sequencing tasks), as well as MoCA were adjusted using the Benjamini–Hochberg procedure controlling the false discovery rate. Statistical significance was determined based on the adjusted p-values (adj p).

## Results

Of 139 at-risk-of-DLB subjects with TCS, 18 did not have sufficient temporal bone window quality (13%). Among the remaining 121 participants, 37 (30.58%) had clearly hyperechogenic SN, 31 (25.62%) displayed mild hyperechogenicity, and 53 (43.80%) showed no hyperechogenicity. For our further analyses, we classified subjects with clearly and mildly hyperechogenic SN as SN+ and those with no hyperechogenicity as SN−. In the SN+ group (*n* = 68), the mean SN area 0.21 ± 0.04 cm², there were 37 men and 31 women, and the mean age was 68.5 ± 7.0 years. In the SN− group (*n* = 53), the mean SN area 0.11 ± 0.03 cm², there were 18 men and 35 women, and the mean age was 66.5 ± 6.9 years. The SN+ and SN− groups did not differ significantly in age (*p* = 0.1155). However, sex distribution differed significantly between groups (*p* = 0.0250), see also the Methods section. The distribution of diagnostic categories did not differ significantly between the SN+ (HC = 9, CCF + = 30, CCF−/MCI + = 7, aMCI-LB = 22) and SN− (HC = 16, CCF + = 16, CCF−/MCI + = 6, aMCI-LB = 15) groups (*p* = 0.1190), as indicated by the chi-square test of independence.

The results of all clinical scores are shown in Table 1.

**Table 1:**
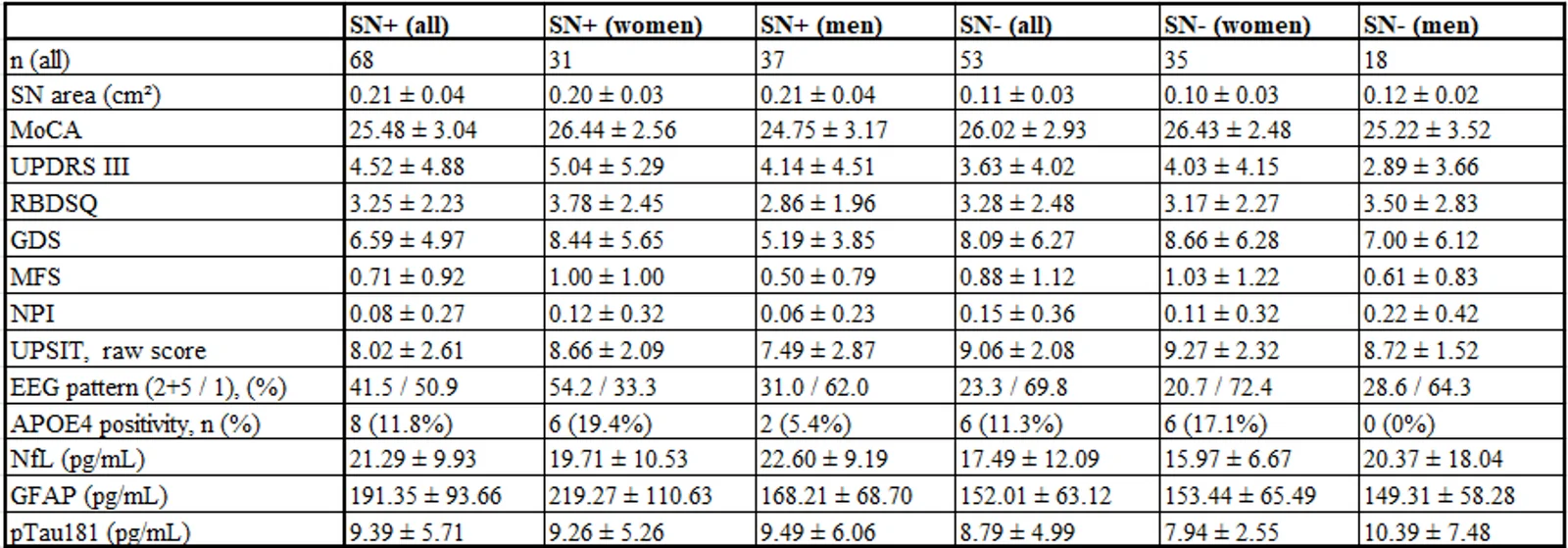
Clinical, cognitive, EEG, and plasma biomarker characteristics stratified by substantia nigra echogenicity and sex

Data are presented as mean ± SD

*SN+* substantia nigra hyperechogenicity,* SN−* substantia nigra normoechogenicity,* MoCA* Montreal Cognitive Assessment,* UPDRS III* Unified Parkinson’s Disease Rating Scale part III,* RBDSQ* REM Sleep Behavior Disorder Screening Questionnaire,* GDS* Geriatric Depression Scale,* MFS* Mayo Fluctuations Scale,* NPI* Neuropsychiatric Inventory, * UPSIT* University of Pennsylvania Smell Identification Test,* EEG* electroencephalography,* APOE4* apolipoprotein E ε4 allele,* NfL* neurofilament light chain,* GFAP* glial fibrillary acidic protein,* pTau181* phosphorylated tau at threonine 181

### Core clinical features

The mean UPDRS III score was 4.5 ± 4.9 in the SN+ group (5.0 ± 5.3 in women and 4.1 ± 4.5 in men) and 3.6 ± 4 in the SN− group (4.0 ± 4.2 in women and 2.9 ± 3.7 in men). No significant difference was observed in the UPDRS III scores between the SN+ and SN− groups (*p* = 0.2011), nor specifically for women (*p* = 0.3118) or men (*p* = 0.0965) separately. No significant differences were observed between the SN+ and SN− groups in RBD screening (*p* = 0.7544), nor specifically for women (*p* = 0.3078) or men (*p* = 0.6797) separately; neuropsychiatric symptoms assessed by the NPI (*p* = 0.2723), sex-stratified analyses likewise showed no significant differences in women (*p* = 0.0538) or men (*p* = 0.1508). Similarly, no group differences were detected in the MFS (*p=*0.9628); this finding remained non-significant in both women (*p=*0.6640) and men (*p=*0.7205).

### Cognitive outcomes

As for the global cognitive impairment, the mean MoCA score in the SN+ group was 25.5 ± 3 (26.4 ± 2.6 in women and 24.8 ± 3.2 in men); in the SN− group, it was 26.0 ± 2.9 (26.4 ± 2.5 in women and 25.2 ± 3.5 in men). Statistical analyses showed no significant differences either between SN+ and SN− groups (adj *p* = 0.4938), or separately in women (adj *p* = 0.9633) and men (adj *p* = 0.7704). Across verbal memory measures, SN+ subjects performed significantly worse on the PVLT, including immediate recall (adj *p* = 0.0384), short-delay recall (adj *p* = 0.0384), and long-delay recall (adj *p* = 0.0384). All three effects were driven by males (adj *p* = 0.0064, adj *p* = 0.0012, and adj *p* = 0.0163, respectively); no significant differences were found among females (adj *p* = 0.8387). No group differences were observed in visual memory as assessed by the BVMT (all adj *p* > 0.3). Regarding attention/ executive functions, no differences were observed between the groups as measured by the DSST (adj *p* = 0.9516), Letter-Number Sequencing (adj *p* = 0.8389) and Picture Arrangement performance (adj *p* = 0.6343). No significant differences were observed between SN+ and SN− participants in semantic verbal fluency (adj *p* = 0.0928), and this remained non-significant after stratification by sex (women: adj *p* = 0.9471; men: adj *p* = 0.2123). Similarly, lexical verbal fluency did not differ between groups (adj *p* = 0.3476), with no significant differences in either women (adj *p* = 0.9471) or men (adj *p* = 0.5705). Regarding the visuo-constructive functions, the median Shannon entropy for the PCT non-significantly differed between the groups in the global on-surface condition (*p* = 0.0574). Sex-stratified analyses revealed no significant difference in women (*p* = 0.4484); a significant difference was observed in men (*p* = 0.0321).

For the results of all selected tests, see Table 2.

**Table 2:**
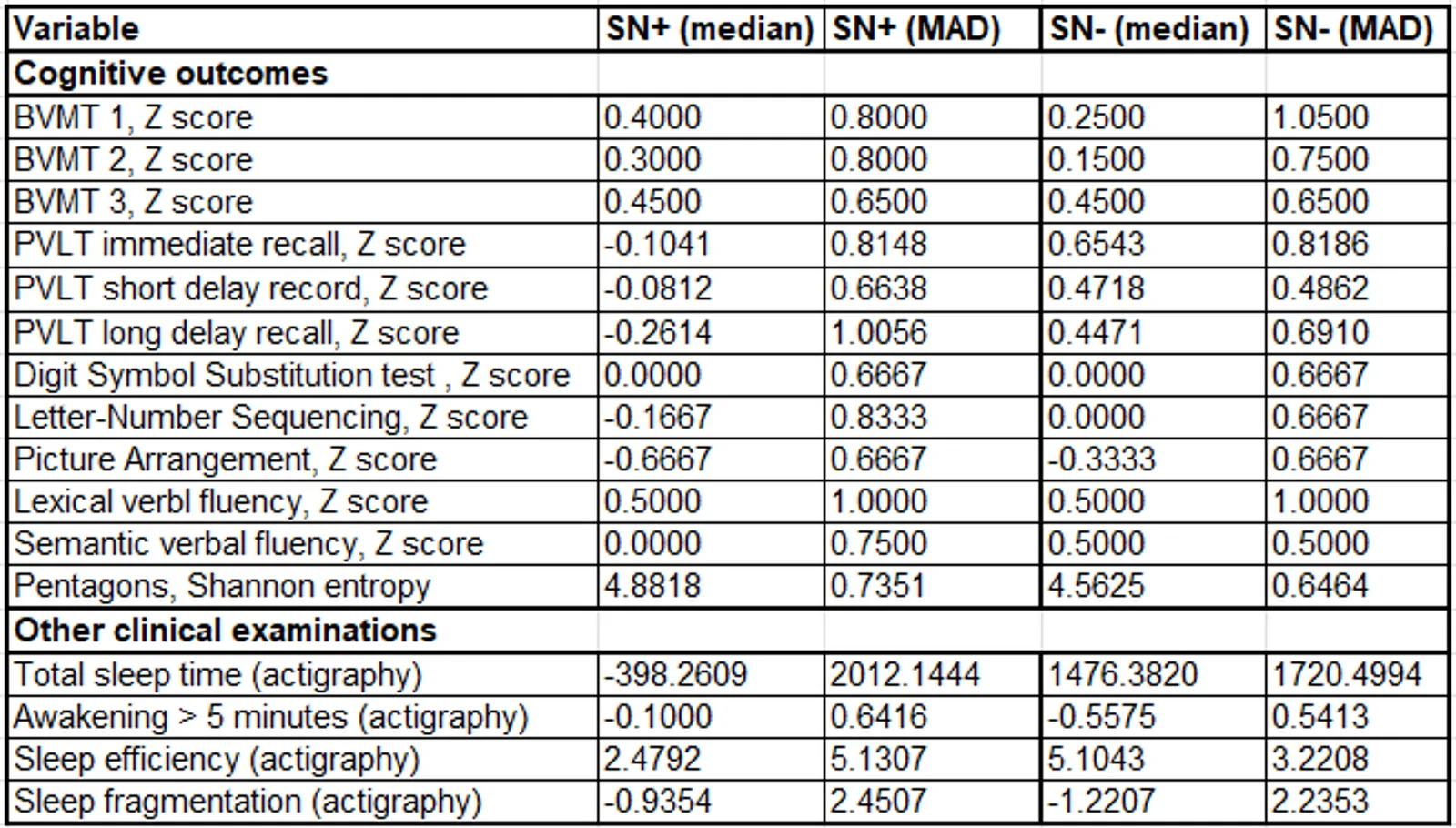
Z-scores of selected cognitive measures and descriptive statistics of tablet-based and actigraphy-derived assessments stratified by substantia nigra echogenicity

Data are presented as median and median absolute deviation (MAD)

*SN+* substantia nigra hyperechogenicity,* SN−* substantia nigra normoechogenicity,* BVMT* Brief Visuospatial Memory Test,* PVLT* Philadelphia Verbal Learning Test

### Other clinical features

In actigraphy-derived sleep parameters, significant group differences persisted after correction for multiple comparisons. Total sleep time differed between SN+ and SN− participants (adj *p* = 0.0187). Similarly, the number of awakenings lasting longer than 5 min (adj *p* = 0.0443) and sleep efficiency (adj *p* = 0.0443) were significantly different between groups. However, sex-stratified analyses did not reveal significant differences after correction in either women (total sleep time: adj *p* = 0.1507; awakenings > 5 min: adj *p* = 0.3661; sleep efficiency: adj *p* = 0.3794) or men (all adjusted *p* ≥ 0.3981). Sleep fragmentation did not differ between groups in the overall analysis (adj *p* = 0.5651) nor after stratification by sex. Depressive symptoms assessed using the GDS did not differ significantly between SN+ and SN− participants in the overall analysis (*p* = 0.2672). Likewise, no significant differences were observed after stratification by sex (women: *p* = 0.8693; men: *p* = 0.4686). Olfactory function assessed by the University of Pennsylvania Smell Identification Test (UPSIT) percentile score did not differ significantly between SN+ and SN− participants in the overall analysis (*p* = 0.2153); no significant differences were observed after stratification by sex (women: *p* = 0.4658; men: *p* = 0.2177).

### Paraclinical examinations (EEG and plasma biomarkers)

In our cohort, all five EEG patterns were identified. Specifically, pattern 1 was observed in 74 participants, pattern 2 in 16, pattern 3 in 7, pattern 4 in 3, and pattern 5 in 23 participants. EEG pattern analysis (presence of DLB-related patterns 2 and 5 versus normal pattern 1) revealed a significant difference between SN+ and SN− participants in the overall analysis (*p* = 0.0109). In the SN+ group, the mean proportion of patterns 2 + 5 was 41.5%, compared to 23.3% in the SN− group, whereas pattern 1 was more frequent in SN− participants (69.8%) than in SN+ participants (50.9%). Sex-stratified analysis demonstrated a significant difference among women (*p* = 0.0059). SN+ women showed a markedly higher proportion of pattern 2 + 5 compared to SN− women (54.2% vs. 20.7%) and a lower proportion of pattern 1 (33.3% vs. 72.4%). In men, no significant difference was observed (*p* = 0.4989). The proportion of patterns 2 + 5 was similar between SN+ and SN − men (31.0% vs. 28.6%), as was pattern 1 (62.0% vs. 64.3%).

APOE4 positivity did not differ significantly between SN+ and SN− participants in the overall analysis (*p* = 0.2916), nor after sex stratification (women: *p* = 0.0556; men: *p* = 0.2509). APOE4 positivity was present in 8/68 (11.8%) SN+ participants and 6/53 (11.3%) SN− participants. Among women, APOE4 positivity was detected in 6/31 (19.4%) SN+ and 6/35 (17.1%) SN− participants. Among men, 2/37 (5.4%) SN+ participants were APOE4-positive; none of the SN− men (0/18) carried APOE4. Neurofilament light chain (NfL) levels differed significantly between SN+ and SN − in the overall analysis (*p* = 0.0323). Mean NfL concentration was higher in SN+ than in SN− participants (21.29 ± 9.93 pg/mL vs. 17.49 ± 12.09 pg/mL). Sex-stratified analysis revealed a significant difference in men (*p* = 0.0104), but not in women (*p* = 0.4198). In men, NfL levels were 22.60 ± 9.19 pg/mL (SN+) and 20.37 ± 18.04 pg/mL (SN−). In women, NfL levels were 19.71 ± 10.53 pg/mL (SN+) and 15,97 ± 6.67 pg/mL (SN−). Glial fibrillary acidic protein (GFAP) did not differ significantly in the overall analysis (*p* = 0.1855). Mean GFAP concentration was 191.35 ± 93.66 pg/mL in SN+ and 152.01 ± 63.12 pg/mL in SN− participants. A significant difference was observed among women (*p* = 0.0420), but not among men (*p* = 0.5049). Among women, GFAP levels were higher in SN+ compared to SN− (219.27 ± 110.63 pg/mL vs. 153.44 ± 65.49 pg/mL). Among men, GFAP levels were 168.21 ± 68.7 pg/mL (SN+) and 149.31 ± 58.28 pg/mL (SN−). Plasma pTau181 levels did not differ significantly between SN+ and SN− participants in the overall analysis (*p* = 0.9358) nor after stratification by sex (women: *p* = 0.7459; men: *p* = 0.5416). Mean pTau181 concentrations were 9.39 ± 5.71 pg/mL in SN+ and 8.79 ± 5.00 pg/mL in SN− participants. Among women, values were 9.26 ± 5.26 pg/mL (SN+) and 7.94 ± 2.55 pg/mL (SN−). Among men, values were 9.49 ± 6.06 pg/mL (SN+) and 10.39 ± 7.48 pg/mL (SN−).

For all the p-values of the selected tests, see Table 3.

**Table 3:**
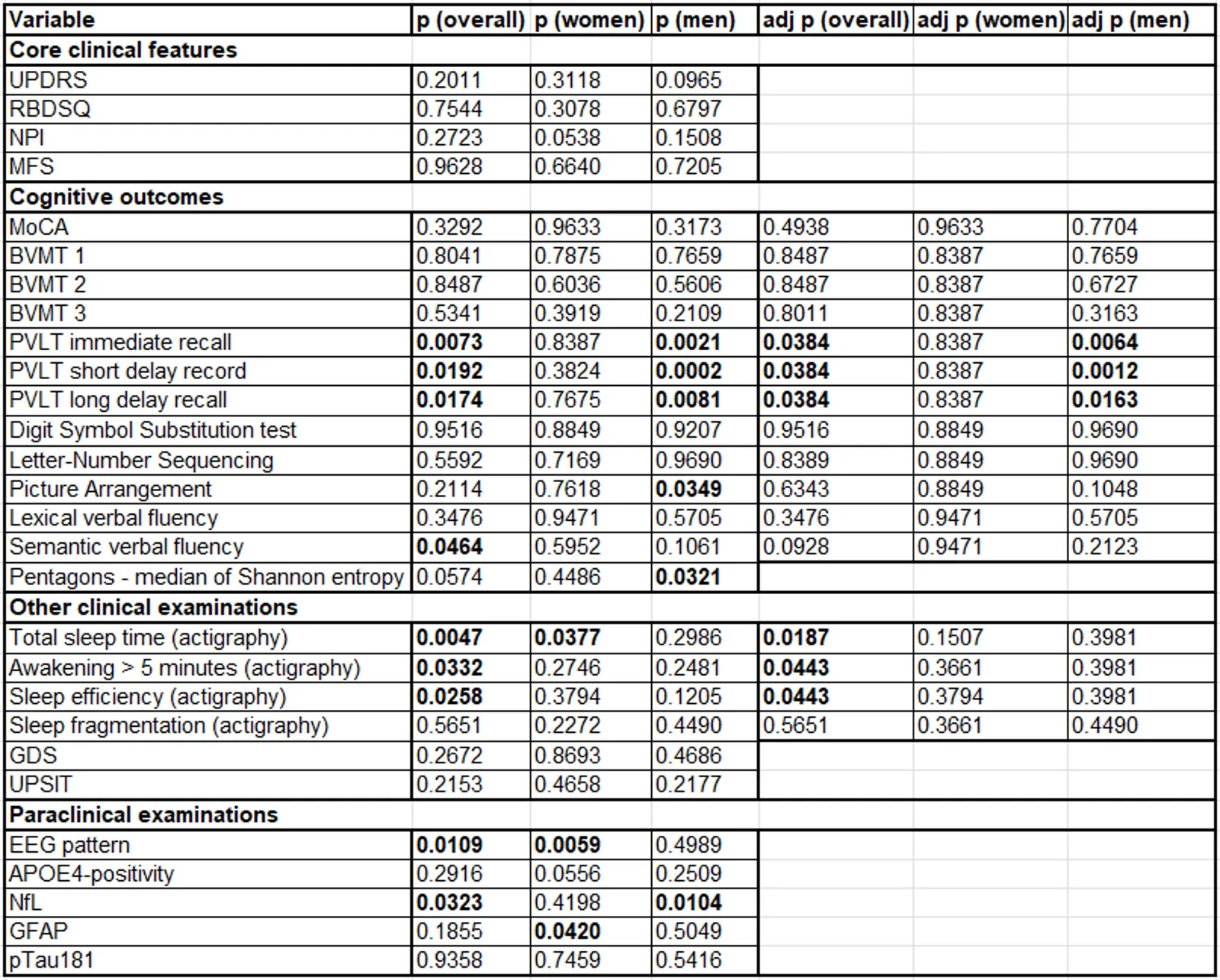
Group comparisons between SN+ and SN− participants: p-values and adjusted p values (overall and stratified by sex)

*Adj p* adjusted p values,* UPDRS III* Unified Parkinson’s Disease Rating Scale part III,* RBDSQ* REM Sleep Behavior Disorder Screening Questionnaire,* NPI* Neuropsychiatric Inventory,* MFS* Mayo Fluctuations Scale,* MoCA* Montreal Cognitive Assessment,* BVMT * Brief Visuospatial Memory Test,* PVLT* Philadelphia Verbal Learning Test,* GDS* Geriatric Depression Scale,* UPSIT* University of Pennsylvania Smell Identification Test,* EEG* electroencephalography,* APOE4* apolipoprotein E ε4 allele,* NfL* neurofilament light chain,* GFAP * glial fibrillary acidic protein,* pTau181* phosphorylated tau at threonine 181

P-values were calculated using the Mann-Whitney U test

Sex-stratified analyses were performed separately in women and men. Adjusted p-values were calculated using the Benjamini-Hochberg false discovery rate (FDR) correction across all variables. Significant results are shown in bold

## Discussion

We evaluated differences in clinical and cognitive features between SN+ and SN− subjects in a unique well-described longitudinal cohort of 121 out of 139 at-risk-of-DLB subjects who had a good quality temporal window for TCS assessment.

Overall, SN+ status (as compared to SN−) was associated with abnormal DLB-specific EEG patterns; sleep disturbances as depicted by altered total sleep time, number of awakenings, and sleep efficiency; elevated NfL levels; and poorer cognitive outcomes particularly in verbal memory and visuo-constructive performance.

The relationship between SN+ and global cognitive performance remains inconsistent in the literature. Some studies report lower MoCA scores in SN+ patients and a negative correlation between SN echogenicity and global cognition (Yu et al. 2018); others found no differences between SN+ and SN− groups (Zhu et al. 2022; Wei et al. 2023). In our cohort, MoCA scores were comparable between groups, suggesting that SN+ alone may not reflect global cognitive decline, at least in subjects with little to no cognitive impairment. The discrepancy may be therefore attributed to differences in the study populations and in the MoCA scores (which were lower in previous studies than in our at-risk-of-DLB subjects). As mentioned above, we found associations with some cognitive test outcomes, namely with visuo-constructive deficits that are typically impaired in MCI-LB (Phillips et al. 2022), but also in verbal memory, which is characteristic of Alzheimer’s disease-related cognitive profiles (Fernández Arias et al. 2023) and is also common in DLB (Ting et al. 2023). Notably, the effects were sex-specific (results driven by males). Although studies have shown differences in prodromal DLB between males and females, particularly in the presence of core clinical features (more frequent in males than females) (Wyman-Chick et al. 2025b; Tsai et al. 2026), further studies are needed to identify distinct sex-dependent cognitive profiles in prodromal DLB. Previous studies have similarly described worse verbal memory in SN+ groups of healthy elderly individuals (Liepelt et al. 2008; Yilmaz et al. 2016a), although inconsistent findings have been also reported (Yilmaz et al. 2016b) in SN+ healthy individuals. While verbal memory impairment may be related to AD comorbidity which is common in DLB even in prodromal cases (McKeith et al. 2017, 2020), our SN+ group did not significantly differ from the SN− group with regard to pTau181 plasma values, i.e. a marker of the magnitude of brain β-amyloid pathology (De Meyer et al. 2024). The APOE4 prevalence did not differ between the two groups either. Reduced verbal fluency has been described in an SN+ cohort of depressive individuals without any parkinsonian symptoms (Hoeppner et al. 2009) and in an SN+ group of healthy elderly individuals (Liepelt et al. 2008); other studies found no association with semantic fluency in healthy elderly SN+ individuals (Yilmaz et al. 2016a). Our results indicated mildly reduced semantic fluency in SN+ participants; lexical fluency remained unaffected, suggesting a specific executive dysfunction rather than a broader language deficit. However, this result has to be taken with caution as it did not survive correction for multiple comparisons. Executive dysfunction has been inconsistently reported in SN+, with some studies demonstrating impairments, particularly in the Trail-Making Test part B (Yilmaz et al. 2016b), and others showing nonsignificant results (Liepelt et al. 2008; Hoeppner et al. 2009). Although overall Picture Arrangement (PAT) performance in our cohort did not differ between groups, SN+ males performed significantly worse. PAT has been widely used to assess higher-order cognitive functions, specifically context-dependent reasoning, and has been used to assess executive functions in studies involving subjects in the DLB spectrum (Bohnen et al. 2010; Kotagal et al. 2012). However, this result did not survive correction for multiple comparisons either. Consistent with prior reports indicating no association between SN echogenicity and visuospatial memory (Liepelt et al. 2008; Yu et al. 2018), we found no differences in the BVMT. On the other hand, we found differences in the performance on the digitized PCT. This task has been shown to assess both visuo-construction (i.e. a posterior cortical cognitive function) and executive functions, and predict cognitive decline in PD (Williams-Gray et al. 2013). In our previous study in early PD patients without any clinically manifested cognitive decline, mean Shannon entropy was correlated with the gray matter volume variability of the superior parietal lobule, i.e. a posterior cortical region engaged in both visual attention and visual-spatial processing (Brabenec et al. 2022).

In accordance with most studies, our results showed no relationship between SN echogenicity and UPDRS III motor scores (Behnke et al. 2013; Vilas et al. 2015; Li et al. 2015; Jesus-Ribeiro et al. 2016; Favaretto et al. 2016; Yu et al. 2018; Miyamoto and Miyamoto 2020) suggesting that SN+ alone may not reflect clinically manifested motor dysfunction. Notably, results in PD remain inconclusive; some authors were able to relate motor symptom severity to SN echogenicity (Fu et al. 2022). Our result in the at-risk-of DLB cohort is not surprising bearing in mind that our participants had only subtle symptoms of parkinsonism if any. Regarding other core clinical symptoms of DLB, we did not find any clear association of SN echogenicity with RBD symptoms, hallucinations, or fluctuation of cognition as assessed by validated scales. RBD is considered one of the most robust prodromal markers of synucleinopathies, and several studies have reported an association between increased substantia nigra echogenicity and the presence of RBD (Stockner et al. 2009; Iwanami et al. 2010; Shin et al. 2013; Li et al. 2017; Iranzo et al. 2017). Furthermore, higher SN echogenicity has been observed in individuals with isolated RBD compared to controls, although SN+ was present only in a minority of cases (Mašková et al. 2020). In line with the latter notion, we found no differences between SN+ and SN− groups with regard to symptoms of RBD as assessed by the screening questionnaire (but not polysomnography), either in the overall analysis or after stratification by sex. As for hallucinations, the relationship between SN+ and visual hallucinations remains unclear. Some studies have reported no association between SN echogenicity and hallucinations across patients with dementia with Lewy bodies, Alzheimer’s disease, and healthy controls (Favaretto et al. 2016); other studies have demonstrated a significantly larger SN echogenic area in Parkinson’s disease patients experiencing visual hallucinations (Li et al. 2020; Del Toro Pérez et al. 2021; Wei et al. 2023). These discrepancies likely reflect differences in study populations and disease stages. In our cohort, no significant differences were observed between SN+ and SN− groups in the NPI hallucination subscores. Notably, explanation for the negative findings may be the very low prevalence of hallucinations in our cohort, which substantially limits the statistical power to detect potential associations with SN+. Previous transcranial sonography studies have rarely evaluated fluctuation severity using standardized instruments such as the MFS. In our cohort, no association was observed between SN+ and fluctuation scores, either in the overall analysis or after stratification by sex. The absence of association with SN echogenicity may be linked to the fact that SN echogenicity is a static “trait” marker of SN vulnerability rather than a dynamic marker of behavioral temporal changes.

In line with the literature, sleep disturbances represent a distinctive clinical feature of DLB, including poor subjective sleep quality and excessive daytime sleepiness, in addition to RBD (Elder et al. 2022). Although our wearable does not diagnose RBD directly, it captures sleep-wake and movement-derived measures. We included four actigraphy metrics previously used in RBD screening, namely total sleep time, awakenings longer than 5 min, sleep efficiency, and sleep fragmentation (Filardi et al. 2020; Brink-Kjaer et al. 2023; Šandala et al. 2025). In this context, the lower total sleep time, higher number of awakenings longer than 5 min, and lower sleep efficiency observed in SN+ individuals are broadly consistent with the more fragmented and less efficient sleep reported in RBD cohorts (Filardi et al. 2020; Šandala et al. 2025). However, the specificity of these actigraphic measures remains limited because similar patterns may also occur in other sleep-motor disorders (Stefani et al. 2018).

While some studies have linked SN+ to a higher incidence of depression in both PD and non-PD populations (Walter et al. 2007; Behnke et al. 2010; Del Toro Pérez et al. 2021), others have failed to establish a significant relationship (Vilas et al. 2015; Bouwmans et al. 2016; Yu et al. 2018; Şenel et al. 2020; Zhu et al. 2022; Wei et al. 2023). In our cohort, depressive symptoms measured by GDS did not significantly differ between SN+ and SN− individuals, either in the overall analysis or after stratification by sex. Our participants also did not meet criteria for major depressive episodes; as assessed by GDS, they had mostly subtle depressive symptoms.

In contrast to previous literature, which reported a relationship between SN+ and reduced olfactory function in PD and even in premotor PD (Behnke et al. 2010; Berg et al. 2010; Liepelt et al. 2011; Richter et al. 2017), we did not observe any difference between SN+ and SN− with regard to olfactory function. In our cohort, SN+ individuals exhibited numerically lower smell test scores than SN− participants, indicating a trend toward worse olfactory performance; however, the difference did not reach statistical significance, even after sex stratification. Our result accords with other studies that found no significant connection (Miyamoto and Miyamoto 2020; Wei et al. 2023). Notably, clear hyposmia was very rare in our at-risk-of-DLB cohort—it was present in only 7 participants. This accords well with the DLB body-first hypothesis (Turcano et al. 2022).

Regarding the plasma markers, we observed increased NfL levels in SN+ as compared to the SN− group and these results were clearly driven by male sex. Higher NfL levels (a marker of neurodegeneration and axonal loss) were associated with lower MMSE scores and faster cognitive decline in DLB (Bolsewig et al. 2024). In accordance with our result, previous research has shown a higher likelihood of cortical atrophy in males than females in a large cohort from the European-DLB consortium and Mayo Clinic (Oltra et al. 2023).

Finally, and totally novel, we were able to relate SN echogenicity with EEG patterns characteristic of DLB. These patterns are mostly based on a lower presence of alpha dominant activity as well as on an increased presence of dominant theta/delta activity in the posterior cortical electrodes of resting-state EEG (Bonanni et al. 2008; Mahajan et al. 2025). The difference was more pronounced in females than in males. Interestingly, the basal forebrain cholinergic system (especially the nucleus basalis of Meynert; NBM), rather than dopaminergic cell loss in SN, plays a key role in the generation and stabilization of posterior dominant alpha rhythms (Sharma and Nadkarni 2020). The cholinergic system is also reflected in posterior cortical cognitive deficits like those in verbal memory and drawing of intersecting pentagons (Gratwicke et al. 2015; Woo et al. 2025), which were found to be linked with SN echogenicity in our study. Taken together, it seems that SN+, a trait marker of synucleinopathy, may be related to early cholinergic deficits and axonal loss as a marker of early neurodegeneration in our at-risk-of-DLB cohort. While SN+ reflects iron deposition and microglial activation rather than dopaminergic deficits (Berg 2006; Desai Bradaric et al. 2012; Zhu et al. 2017), we may speculate that this marker of disease vulnerability may be related to early cholinergic deficits and axonal loss, which are typical for the prodromal stages of DLB (Kantarci et al. 2022; Jellinger 2023; Okkels et al. 2024; Woo et al. 2025). Although plausible, we have to stress that we did not directly study cholinergic deficits using imaging methods and therefore this speculation has to be taken with caution. Longitudinal studies with cholinergic PET tracers or MRI evaluating NBM and cholinergic white matter integrity may provide further support of this notion and further studies are needed to shed more light on the sex-differences observed in our study.

Study limitations: Our at-risk-of-DLB subjects are characterized clinically; no suggestive biomarkers of DLB were used such as dopaminergic imaging or polysomnography. Of note, dopaminergic imaging has low sensitivity in prodromal DLB (McKeith et al. 2020) and neither polysomnography nor α-synuclein examination using seed amplification assay from CSF or skin biopsy are broadly available in our country. An additional limitation of our study is its cross-sectional design, which does not allow us to draw conclusions about the predictive value of SN hyperechogenicity for the future development of DLB. Longitudinal studies will be necessary to further explore these associations and to clarify their possible prognostic relevance.

## Conclusion

Taken together, we found that SN echogenicity in our at-risk-of-DLB cohort was linked to a plasma marker of axonal loss and DLB-related EEG patterns and posterior cortical cognitive deficits, both of which have been indirect markers of cholinergic denervation characteristic of early phases of DLB. Some of these results differed between males and females; further research is needed to replicate and explain these sex-specific differences.

## Data Availability

The data supporting the findings of this study are not openly available. Anonymized data are, however, available from the corresponding author upon request.
